# Topical Formulations of Serratiopeptidase: Development and Pharmacodynamic Evaluation

**DOI:** 10.4103/0250-474X.62246

**Published:** 2010

**Authors:** N. M. Nirale, Mala D. Menon

**Affiliations:** Department of Pharmaceutics, Bombay College of Pharmacy, Kalina, Santacruz (East), Mumbai-400 098, India

**Keywords:** Antiinflammatory, gel, ointment, serratiopeptidase

## Abstract

Serratiopeptidase, an enzyme derived from *Serratia marcescences* strain E-15 (ATCC 21074), present in the gut wall of the silk worm possesses anti-inflammatory properties, and can prove to be a suitable alternative to commonly used non steroidal antiinflammatory agents. Being sensitive to gastric degradation, serratiopeptidase is conventionally given orally in the form of enteric coated tablet formulations. Topical formulations of serratiopeptidase would be useful to treat local inflammations and may prove to be more effective compared to non steroidal antiinflammatory agents. The present study investigates the feasibility of developing topical preparations of serratiopeptidase in the form of ointments and gels. Excipient compatibility of serratiopeptidase with various excipients and polymers, formulation development, characterization and stability studies have been carried out. Stable formulation was evaluated for anti-inflammatory activity by oxazolone induced ear edema method in mice and allergenic potential by passive cutaneous anaphylaxis.

The non-steroidal antiinflammatory drugs (NSAID's) like ketoprofen, diclofenac sodium are widely used for the treatment of acute and chronic arthritic conditions. Oral administration of NSAIDs often causes gastric irritation leading to ulcer and other systemic side effects. Protease enzymes belonging to family metalloprotease, have been successfully tested for their antiinflammatory properties, which include trypsin, chymotrypsin and serratiopeptidse (SRP). SRP, an enzyme derived from *Serratia marcescences* strain E-15 (ATCC 21074), present in the gut wall of the silk worm possesses antiinflammatory properties, and can prove to be a suitable alternative to commonly used NSAIDs.

Formulations of SRP are available mainly in the form of enteric coated tablet (Danzen, Takeda Japan). Conventionally these tablets are dry coated with enteric polymer[1,2]. SRP is reported to undergo acid hydrolysis at gastric pH thereby decreasing the stability in GIT. SRP is given at a dose of 5-10 mg three times a day. In order to increase the stability of SRP (reduction of acid hydrolysis) and improve bioavailability various other approaches of delivering the enzyme at the target site have been reported, which include glyceryl monooleate based systems (cubic phase transformation)[3] and *in situ* gelling systems for intranasal delivery[4]. SRP can actually team up with antibiotics and deliver increased concentrations of antibiotics to the site of the infection. Bacteria can go through a process of producing biofilm, which results in resistance to antibiotics. A study by a team of Italian researchers suggests that proteolytic enzymes such as SRP could significantly enhance the effectiveness of antibiotics against biofilm and can inhibit biofilm formation[5]. Orally given SRP shown to alter viscoelasticity of sputum in patients with chronic airway disease[6]. SRP has been shown to enhance the activity of several antibiotics including ampicillin, ciclacillin, cephalexin, minocylcine and cefotiam. However orally delivered SRP has various side effects, which include anorexia, nausea and GI disturbance. Therefore topical application of enzyme offers potential advantages of delivering the enzyme directly to the site of action[7,8]

Topical formulations of SRP would be useful to treat local inflammations and may prove to be more effective compared to topical NSAIDs. The present study investigates the feasibility of developing topical preparations of SRP in the form of ointments and gels.

## MATERIALS AND METHODS

SRP was obtained from Advanced Enzyme Technology Ltd, Thane as a gift sample. Carbopol 934 obtained from Noveon Inc. USA, hydroxypropylmethylcellulose (HPMC) was obtained from Colorcon Asia Pvt. Ltd. Xanthan gum (Xantural 180) was obtained from Signet Chemical Corporation, Mumbai as a gift sample. Glyceryl monooleate (GMO) was obtained as gift sample from M/s Mohini Organics Pvt. Ltd., Mumbai. Ovalbumine (OVA) Sigma Chemical company, St. Louis, USA. Polyethylene glycol (PEG) 400, 4000 and 6000, and all other chemicals were of analytical grade and purchased from S. D. Fine-Chem Ltd. and Thomas Baker, Mumbai.

### Determination of Proteolytic activity of SRP:

The proteolytic activity was determined as per the method reported in Food Chemical Codex (FCC) 2003[9]. The assay was based on 30 min proteolytic hydrolysis of casein at 37° and pH 7.0. Unhydrolysed casein was removed by filtration and the solubilised casein (tyrosine) was determined spectrophotometrically at 275 nm. In this method enzyme activity (Unit/mg) expressed in terms of One Bacterial Protease Unit (PC) and defined as that quantity of enzyme that produces the equivalent of 1.5 μg/ml of L-tyrosine per minute under the conditions of assay.

Enzyme activity was calculated by using the equation, PC/g=(A_U_/A_S_)×(22/30W), where A_S_ is Absorbance of 1.5 μg/ml of L-tyrosine, A_U_ is absorbance of the sample enzyme, 22 is the final volume of the reaction mixture, W is Weight (g) of the original sample taken.

### Compatibility Studies with Excipients:

This study was conducted using selected excipients at neutral pH. Polymers selected were carbopol 934, HPMC (K4M, K100M, A4M, A15C), and xanthan gum. Other excipients selected were methyl paraben (preservative), propylene glycol (humectant), tween 80, dimethyl sulfoxide, menthol, eucalyptus oil. All the compatibility studies were conducted at room temperature. SRP solution (1% w/v) was added to each polymer dispersion (0.5% w/v). The mixture was stirred continuously (Remi Overhead stirrer), and maintained for 12 h at room temperature.

The proteolytic activity of mixture was determined at zero hour and after 12 h by using proteolytic assay method. Similar study was performed in presence of calcium chloride (1:1 ratio with respect to SRP) as a stabilizer. The inhibition factor (IF)[10] was calculated as per the equation, IF_(Inhibition factor)_ = Activity_(control)_/Activity_(polymer)_.

Similar compatibility experiments were performed with methyl paraben 0.3% w/v solution, propylene glycol 10% w/v solution, tween 80 (1% w/v solution in distilled water), dimethyl sulfoxide (15% v/v in distilled water), menthol, and eucalyptus oil. Menthol and eucalyptus oil were dispersed in propylene glycol in the ratio of 1:1 (v/v). Accurately weighed 10 mg of SRP was mixed with above solutions on cyclomixer for 15 min, and enzyme content was determined by proteolytic method.

### Selection of Medium for Permeation Studies:

Stability of SRP in different media like distilled water, Tris-buffer pH 7.0, phosphate buffer 7.0 was evaluated for a period of 12 h at room temperature as well as at 37°. Accurately weighed 10 mg of SRP was dissolved and diluted to 10 ml with respective media. 0.25 ml of this solution was further diluted to 25 ml with respective media to get test sample solution. These solutions were kept at room temperature and 37° in water bath for 12 h. At predetermined time intervals (0, 2, 4, 6, 8, 10, 12 h), 2 ml samples were withdrawn and proteolytic activity was determined as described earlier.

### *In vitro* permeation studies:

The abdominal skin of guinea pig was excised and the adhering fat and other visceral tissue were removed. The skin was then washed thoroughly with normal saline, and mounted on Franz diffusion cell equipped with 16.5 ml of Tris-buffer (pH 7.0) in the receptor compartment, temperature was maintained at 37±1°. One milliliter of 5 mg/ml solution of SRP was placed onto the skin surface. The receptor fluid was constantly stirred with a small bar magnet. At predetermined time intervals, 1 ml samples were withdrawn and replaced with equal amount of fresh Tris-buffer pH 7.0. Each withdrawn aliquot was analyzed for enzyme content and permeability was calculated. Various permeation enhancers like dimethyl sulfoxide (DMSO) (10-99%), isopropyl myristate (IPM) (10-99%), menthol, HPβCD (10% in distilled water), eucalyptus oil (50% in PG), oleic acid (10% in PG), Transcutol-P (10% in distiled water) and N-methyl pyrolidone (10% in distilled water) were evaluated.

### Formulation of xanthan gum gel (XG gel):

Specified amounts of methyl paraben, Tris-buffer, propylene glycol, calcium chloride were dissolved in half the quantity of distilled water under mechanical stirring. Xanthan gum was slowly sprinkled over to this solution, polymer was allowed to swell for 30 min. SRP was dissolved in 1/4^th^ quantity of distilled water and mixed with polymer gel under slow stirring. Final weight was made up with distilled water (Table 1).

**TABLE 1:** COMPOSITION OF TOPICAL FORMULATIONS

Ingredients	XG (% w/w)	PEG GMO (% w/w)
Xanthan gum	3	-
Methyl paraben	0.3	-
Propylene glycol	10	-
Tris-buffer	0.03	-
CaCl_2_	1	-
Enzyme(SRP)	1	-
Distilled Water	q.s. to 100	-
GMO (Monegyl 0100)	-	10
PEG 400	-	40
Propylene glycol	-	10
SRP	-	1
PEG 4000	-	qs to 100

XG- Xanthan gum gel, PEG GMO- Polyethylene glycol glyceryl monooleate ointment

### Formulation of polyethylene glycol-glyceryl monooleate (PEG-GMO) ointment:

Specified quantities of melted GMO and PEG 400 were gently stirred in a beaker. Specified quantity of SRP was slowly added to this mixture under slow stirring (Part A). Melted PEG 4000 was added to part A under slow agitation until the mixture congealed (Table 1).

### Characterization of prepared formulations:

The prepared gel and ointment formulations were evaluated for pH, rheology, enzyme content, *in vitro* release characteristics and stability. pH measurements were done using digital pH meter (Universal Enterprises, Mumbai), rheological measurements on a Brookfield Synchroelectric viscometer USA, Model RVT spindle numbers 6 and 7.

### Enzyme content analysis:

Enzyme content analysis was performed as per method reported in FCC 2003. Accurately weighed quantity of formulation equivalent to 10 mg of SRP was mixed and dispersed with 5 ml of the Tris-buffer (pH 7.0) in test tube on a cyclomixer for 1 min, and the volume of mixture made up to 10 ml with Tris-buffer (pH 7.0). This solution was suitably diluted with Tris-buffer (pH 7.0), and 2 ml of this diluted solution was used in the proteolytic activity method. Similar study was performed on blank formulation.

### *In vitro* diffusion studies:

The diffusion studies of the prepared gels were carried out in Franz-Diffusion cells using parchment paper. Parchment paper was soaked overnight in Tris-buffer pH 7.0 and dried at room temperature. Parchment paper was mounted on Franz-diffusion cell equipped with 16.5 ml of Tris-buffer pH 7.0 in receptor compartment, temperature was maintained at 37±1°. Gel and ointment formulations (equivalent to 10 mg of SRP) were placed on the parchment paper. The receptor fluid was constantly stirred with a small bar magnet. At predetermined time intervals, 1 ml samples were withdrawn and replaced with equal amount of fresh Tris-buffer pH 7.0. Each withdrawn aliquot was analyzed for enzyme content by proteolytic method.

### *In vivo* studies:

Antiinflammatory activity was done for evaluation of the formulation. Necessary approval from IAEC of Bombay College of Pharmacy was obtained prior to commencement of the experiment. Screening of antiinflammatory activity was carried by oxazolone induced ear edema method in Swiss mice[11]. Mice were sensitized by application of 0.01 ml of 2% solution of oxazolone on the inside of right ear, and left ear remained untreated. After 8 days, under anesthesia all groups were challenged with 0.01 ml of 2% oxazolone solution to inside of the right ear, and left ear remained untreated. After 5 min, 0.1 g of ointment sample and marketed sample (standard) were applied, inside of the right ear of the test groups and standard groups respectively; similarly placebo group received 0.1 g of blank formulation. Twenty four hours later animals were sacrificed and a disc of 5 mm diameter is punched from both ears, and immediately weighed on a balance. Percentage edema inhibition was calculated as, % edema= (Weight of right ear − weight of left ear)/Weight of left ear×100. Mean percentage inhibition and standard deviation were calculated. ANOVA was applied with Tukey's test for multiple comparison between different groups p<0.05 was considered to be statistically significant.

Allergenic potential of the formulation was assessed by passive cutaneous anaphylaxis (PCA) in male Wistar rats and male Swiss mice were used in the study. Wistar rats were bred in-house at the Bombay College of Pharmacy and kept under pathogen free conditions. All procedures performed were in accordance with protocols approved by the IAEC. Six-week-old male Wistar rats (three per group) were epicutaneously sensitized with ovalbumine (OVA, OVA group) or the selected SRP formulation (P42) under ether anesthesia. The back of the rats was shaved with a commercially available depilatory (Veet, Reckitt Benckiser) and electronic razor was used to introduce skin injury. OVA (1 mg/ml) and SRP formulation (1 g) was topically applied to 1×1 cm^2^ patches of sterile gauze, and applied to the respective groups. The gauze was secured on the skin with transparent adhesive tape for 7 days (first sensitization week). On the 14^th^ day (second sensitization week), an identical patch was reapplied to the same skin site of rats. The last epicutaneous sensitization (third sensitization week) was given on the 28^th^ day[12].

After the exposure period (after 28^th^ day), blood was collected from abdominal aorta under urethane anesthesia (1.3 g/kg, i.p.) from all the animals. Serum was separated and pooled from respective treatments and stored at −70° till further analysis to assess IgG and IgE titres. Serial dilutions of pooled serum samples of OVA treated and selected formulation treated animals (1:1 to 1:128) were prepared in saline. Fifty microlitres of OVA (1 mg/ml) in saline was add to 50 μl of serially diluted sera samples and incubated at 37° for 1 h, and observed for coagulation. The highest dilution showing coagulation was noted as IgG titre.

PCA in Swiss mice was used to assess IgE titre[12–14]. The Swiss mice were shaved on the back and intradermal injections (0.05 ml) of the serial dilutions were made on each side of the dorsal skin and the sites were marked. After 48 h, mice were challenged intravenously with 0.5 ml of 0.25% Evans blue solution containing 2 mg/ml of OVA. Thirty minutes later, animals were sacrificed by cervical dislocation; dorsal skin was removed and observed for blue patch at the site of injection. A patch of more than 5 mm diameter was considered as positive reaction for IgE.

## RESULTS AND DISCUSSION

Enzyme replacement therapy has become important in treatment of several metabolic and genetic defects. Lack of adequate delivery or retention of administered enzymes at required sites of action and hypersensitivity reactions are the major limitations in effective therapy. Conventionally SRP is administered as enteric coated oral tablet formulation. Orally administered SRP has systemic side effect, hence to prevent systemic side effect and to enhance local effect there is need to formulate topical delivery systems of the SRP.

The activity of the SRP enzyme as per FCC 2003 was found to be 1972 ± 35.35 Units/mg of SRP. Polyacrylic acid, HPMC, xanthan gum finds application in the pharmaceutical and cosmetic industries as thickening and suspending agents. These polymers are hydrophilic in nature, but the rheological property of polyacrylic acid is pH dependent, and follows the ionization profile of the carboxylic acid functional groups present in the polymer. Leuben *et al.*[10] have reported that these polymers inhibit trypsin, α-chymotrypsin, and carboxypeptidase enzyme activity and this inhibition is pH-dependent. The loss in enzyme activity could be due to the competitive, non-competitive interaction. Many protease enzymes have bivalent cations like zinc and calcium as essential co-factors within their structure[10,15]. SRP has zinc and calcium as co-factors. Its N-terminal proteolytic domain has a catalytic zinc ion (active site), whereas calcium is not essential for its activity but it is necessary to protect it from autolysis[16].

Effect of some common polymers on the proteolytic activity of SRP was evaluated along with Ca^2+^ ions (CaCl_2_) used in 1:1 proportion with SRP in the polymer dispersion. IF (Inhibition Factors) of the polymer in absence of stabilizer as well as its presence is given in Table 2. Polyacrylic acid showed interaction with the stabilizer, IF value was found to be high in all the polymers except xanthan gum when studied in presence of stabilizer. Proteolytic activity of SRP was found to be retained in methyl paraben, propylene glycol, Tween 80 and DMSO, whereas the activities in menthol, eucalyptus oil and oleic acid were found to be reduced (Table 3).

**TABLE 2:** INHIBITION FACTOR OF VARIOUS ENZYMES

Polymer bases	Inhibition Factor in absence of stabilizer		Inhibition Factor in presence of stabilizer ion (CaCl_2_)	
		
	0 h Mean±SD	After 24 h Mean±SD	0 h Mean±SD	After 24 h Mean±SD
Carbopol 934P	1.43±0.01	1.69±0.01	---	---
HPMC (K4M)	1.24±0.01	1.58±0.01	1.26±0.00	1.59±0.008
HPMC (K100M)	1.27±0.02	1.55±0.02	1.25±0.01	1.81±0.037
Xanthan gum	1.12±0.05	1.78±0.02	1.11±0.03	1.14±0.003
HPMC (A4M)	1.10±0.02	2.59±0.01	1.22±0.03	1.83±0.038
HPMC (A15C)	1.12±0.01	1.36±0.02	1.11±0.01	1.74±0.03

SD-Standard Deviation, n=3

**TABLE 3:** COMPATIBILITY STUDIES OF SRP WITH VARIOUS EXCIPIENTS

Ingredients	Percentage activity found Mean± SD
Methyl paraben	90.93±4.04
Propylene glycol	85.86±1.96
Tween 80 (1% w/v solution in distilled water)	107.01±1.80
Dimethyl sulfoxide (15% v/v in distilled water)	85.53±5.31
Menthol	12.82±3.46
Eucalyptus oil	32.84±1.00
Oleic acid	16.26±3.88

SD-Standard Deviation, n=3

**Fig. 1:**
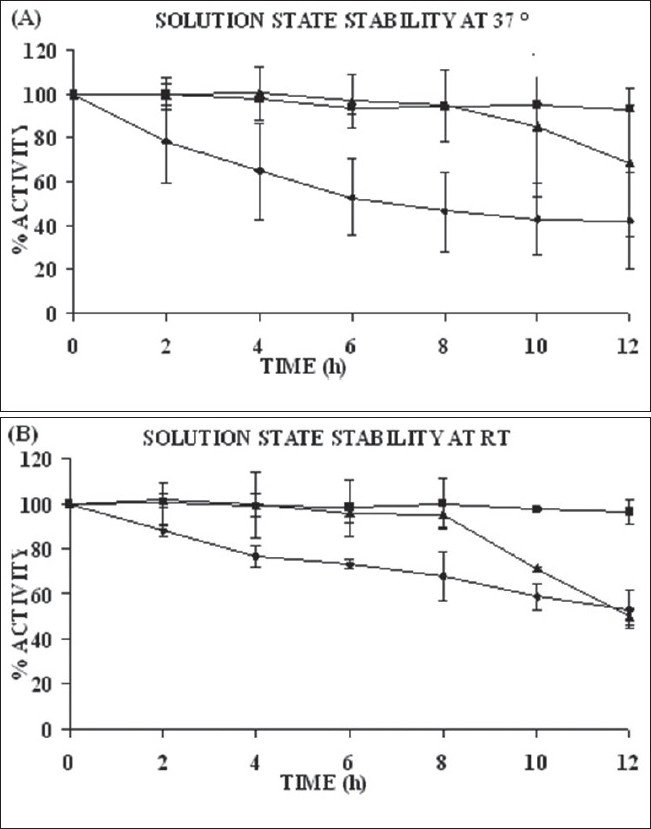
Solution state stability at 37° and at Room temperature The solution state stability was given in % activity of SRP at (A) 37° where, a comparision between distilled water (-◆-), Tris Buffer pH 7.0 (-■-) and phosphate buffer pH 7.0 (-▲-) and (B) at room temperature has been given.

The degree of enzyme hydration is one of the most important factors which can affect enzyme activity and stability in water environment. Biocatalysts are inherently labile and their activity depends on the presence of water molecules, temperature, and pH of the solution. With a view to select suitable dissolution medium for SRP, its stability in different media over a period of 12 h was evaluated. Enzyme activity was determined using proteolytic activity method[17–20]. At room temperature enzyme activity retained in distilled water, Tris-buffer, and phosphate buffer was 53%, 96%, and 49.83% respectively and at 37° enzyme activity retained was 42%, 93%, 68% respectively (fig. 1). Thus Tris-buffer pH 7.0 was found to be the most suitable dissolution medium for prepared SRP formulations.

Enzymes are large molecular weight compounds; therefore permeation through skin is a major issue. Various permeation enhancers like DMSO, IPM, *N*-methylpyrrolidone, oleic acid, transcutol-P, eucalyptus oil, menthol were evaluated in permeation studies. Only IPM and DMSO were found to be effective where as others had negligible effect on the permeation of SRP. However, DMSO and IPM displayed permeation enhancement at concentrations (>50%) which was much higher than their acceptable limits[21].

Carbopol (934P NF) formulation was liquefied on storage at room temperature after one month, so carbopol formulation was not selected for further studies. HPMC gel formulations were not clear in appearance and liquefied on storage at room temperature, hence HPMC gel formulations were not considered for further evaluation xanthan gum at, 3% w/w concentration gave a suitable gel consistency and was selected for further studies. The pH of the gel formulation was adjusted to 7.0 (with Tris-buffer) to stabilize the enzyme. XG gel formulation was found to be faintly yellow coloured, odourless and clear in appearance. The pH of the formulation was found to be between 6.5 to 7.0. Enzyme content was found to be 87.12±1.91%. *In vitro* release profile revealed that 11.72% of the enzyme was permeated at the end of 24 h. Similarly PEG- GMO ointment formulation was found to be colourless with slight characteristic odour and soft opaque in appearance. The pH was found to be 6.52. *In vitro* release study revealed 8.73% of the enzyme SRP was permeated at the end of 24 h. The rheogram of the XG gel as well as PEG-GMO ointment gel showed pseudoplastic flow; with slight degree of thixotropy; and a small yield value was evident.

**Fig. 2:**
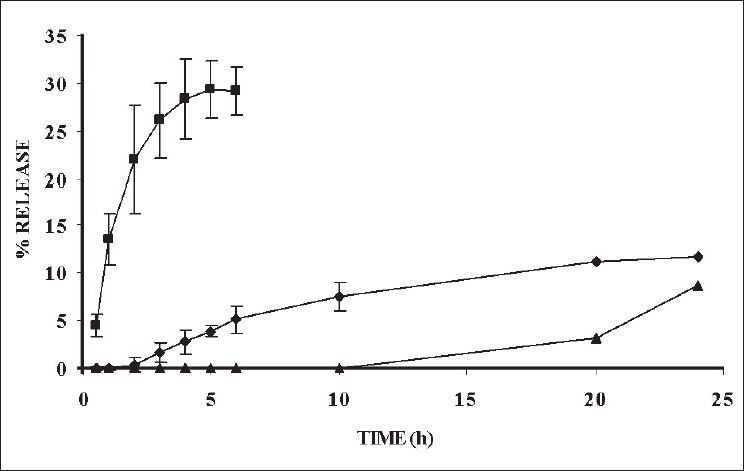
*In vitro* release profile of SRP, XG gel and ointment (P42) formulation Percentage release profile of plain SRP (-■-) and its xanthan gum (XG) gel (-◆-) and polyethylene glycol glyceryl monooleate (PEGGMO) ointment (-▲-)

The *in vitro* release profile of SRP from the various bases (fig. 2) indicated 30% of SRP permeation through parchment paper in 6 h, about 12% of SRP permeated from XG gel in 12 h and only 8.73% of SRP permeated from PEG-GMO ointment. Thus maximum retardation of SRP was seen with PEG-ointment.

XG gel and PEG-GMO were subjected to stability evaluation under 3 conditions viz., refrigerator (4-8°), 25°/60% RH and 40°/75% RH for a period of three months. XG gel batches did not show any marked changes in organoleptic properties during the entire storage period at all storage conditions. However, PEG-GMO formulation showed a slight yellow colouration after 2 month of storage. XG gel formulation exhibited a marked decrease in the pH during the storage period, whereas PEG-GMO showed a minor change in pH. On account of superior stability, PEG-GMO formulation was subjected to *in vivo* evaluation.

**Fig. 3:**
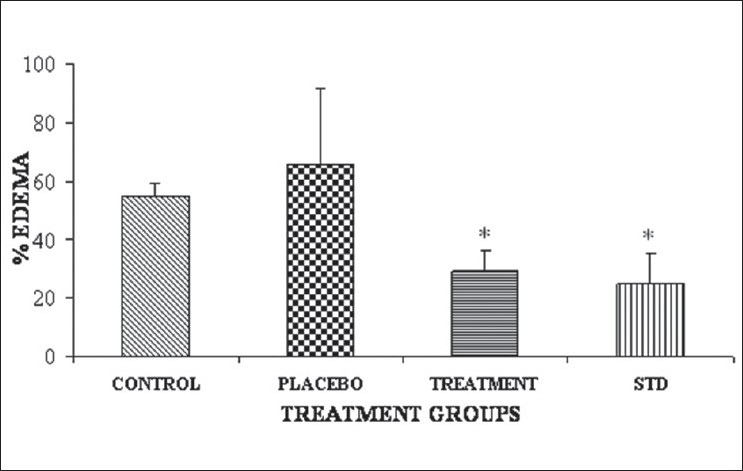
Percent edema after treatment of standard and test ointment formulation Treatment group is polyethylene glycol glyceryl monooleate (PEGGMO) ointment and error bars represents standard deviation (n=6)

Some of the reported methods to induce acute and sub-acute inflammation include UV erythema in guinea pigs, croton oil ear edema in rats and mice, granuloma pouch technique and paw edema method, oxazolone-induced ear edema in mice. The formulation selected for the antiinflammatory study was PEG-GMO ointment as this batch has shown comparable permeability in parchment paper and good stability in comparison to XG gel formulation. The results of the antiinflammatory study are given in fig. 3. PEG-GMO and diclofenac gel (marketed/standard) formulations significantly inhibited ear edema in comparison to control and placebo groups (p<0.05). However, no significant difference between PEG-GMO and diclofenac gel was found (p>0.05).

**Fig. 4:**
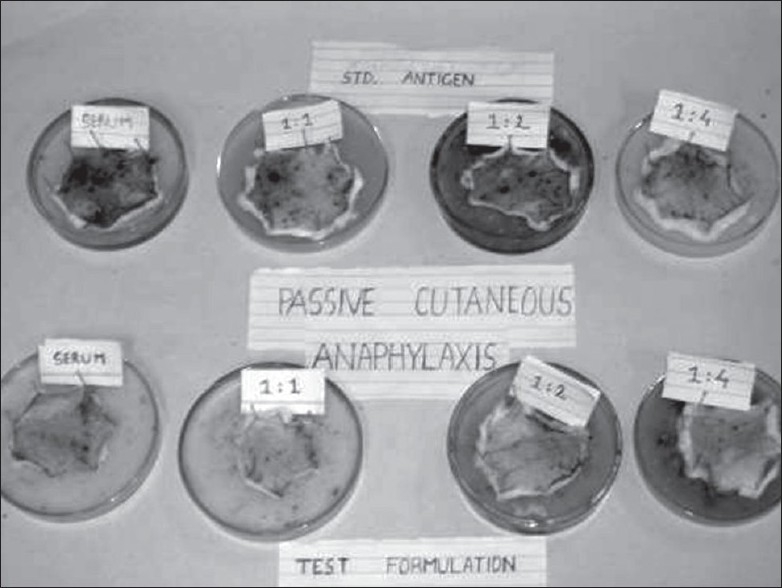
PCA reaction in the standard ovalbumin and test formulations treated albino mice. PCA reaction of Standard ovalbumin antigen in upper row showed dark blue coloured circled patches, Test formulation (polyethylene glycol glyceryl monooleate, PEGGMO ointment) showed considerable reduction in dark circles. The test was carried out with undiluted serum and various dilution ratios of 1:1, 1:2 and 1:4

For the safety evaluation of proteins and peptides, the potential allergenicity of the newly introduced protein(s) has become an important issue. As enzyme replacement therapy involves use of recombinant enzymes as an alternative to the native enzyme, which may be derived from different sources like fungi, bacterial or animal origin, they can act as foreign particles to the host and elicit immune response. Hence, it is necessary to evaluate the developed formulation's potential to produce allergic reactions when applied topically. The IgG titre in serum obtained from OVA-treated Wistar rats was found to be 1:2 as the serum sample showed coagulation at this dilution where as no coagulation was observed in serum of PEG-GMO (1:128 up to dilutions) treated animals. Passive cutaneous anaphylaxis reaction produced by serum from OVA treated rats on Swiss albino mice revealed the IgE antibody titer to be 1:1 where as no PCA reaction was produced by serum obtained from PEG-GMO treated rats (fig. 4). Three mice were used for each dilution level. Low levels of IgG and IgE antibody were found in the OVA treated animals as well as PEG-GMO treated mice.
